# Supplementary material for: Phase transitions in few-monolayer spin ice films
Source: Nat Commun. 2019 Mar 14;10:1219. doi: 10.1038/s41467-019-09187-w (PMC6418252; doi:10.1038/s41467-019-09187-w)
Supplement: Supplementary file 1 — Supplementary Info [file 41467_2019_9187_MOESM1_ESM.pdf]

Supplementary Information for

"Phase Transitions in Few-Monolayer Spin Ice Films"

Bovo et al.

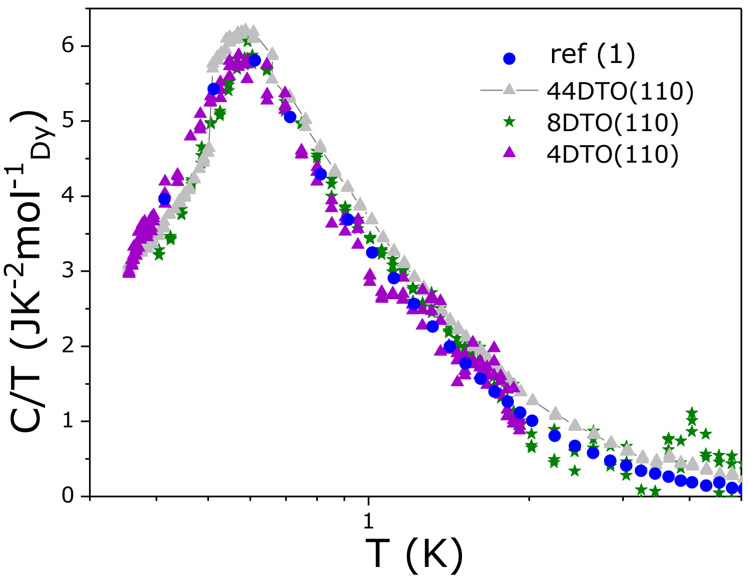

Supplementary Figure 1. Specific Heat for xDTO||YTO(110) series. Colour code, as explained in the legend. Specific heat contribution scales with sample thickness, although the sharp but small discontinuity at $T = 0.510(2)$K visible in x = 44 (grey triangle, with line as guide to the eye) is lost for x = 8 (green stars) and 4 (purple triangle). Our previous result (1) is reported for comparisons; data are in excellent agreement although the old ones (blue circles) fail to detect the discontinuity due to a wider spacing of points.

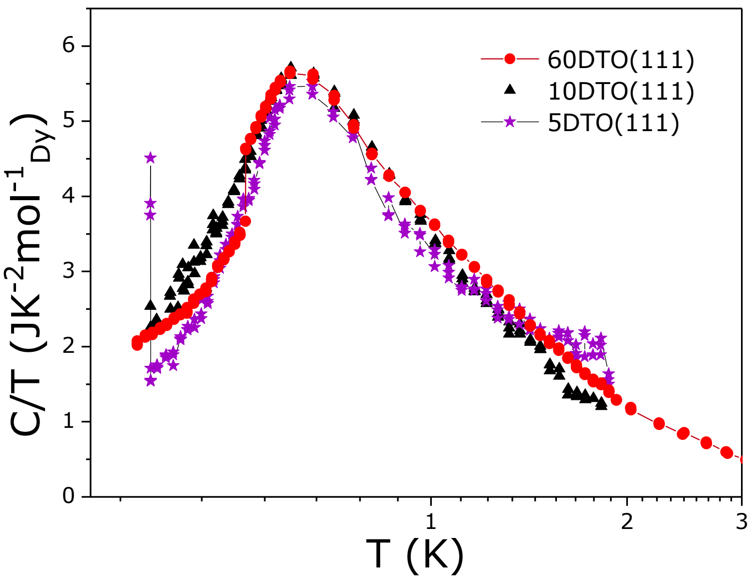

Supplementary Figure 2. Specific Heat for xDTO||YTO(111) series. Colour code, as explained in the legend. Specific heat contribution scales with sample thickness, although the sharp but small discontinuity at $T = 0.520(2)$K visible in x = 60 (red circles, with line as guide to the eye) is lost for x = 10 (black triangle) and 5 (purple stars, with line as guide to the eye). The thinnest sample, x = 5, also shows the hint of a sharp rise at very low temperature.

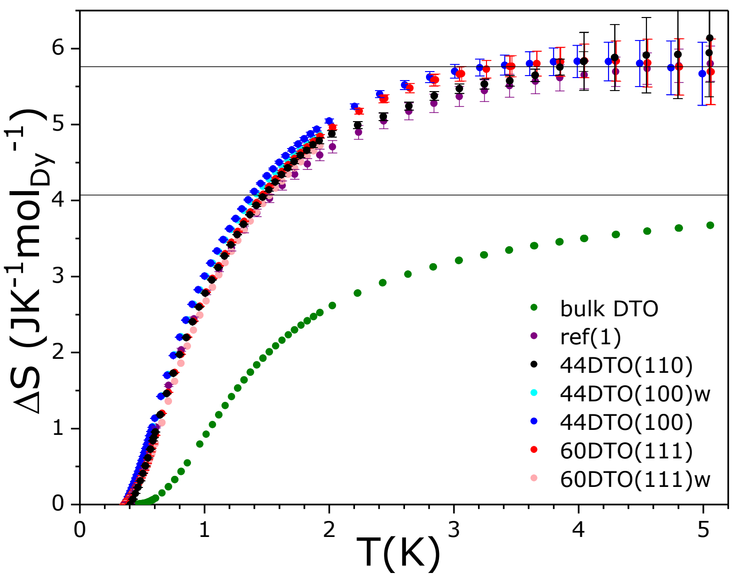

Supplementary Figure 3. Magnetic Entropy curves for DTO||YTO samples. Colour code, as explained in the legend. Magnetic entropy increment found by integrating c/T between 0.35K and 5K for samples reported in the legend. Error bars represent estimated maximum systematic error. Top line corresponds to the expected value for the full spin entropy of $R(ln\ 2)$. The bottom line corresponds to the expected value for a spin ice compound of $R(ln\ 2 - (1/2)\ ln(3/2))$, where the right-hand term is the Pauling residual entropy.

Supplementary Reference

(1) L. Bovo et al., Restoration of The Third Law in Spin Ice Thin Films. *Nature Comms.* **5**, 3439 (2014).
